# Unique Phrenic Nerve-Sparing Regional Anesthetic Technique for Pain Management after Shoulder Surgery

**DOI:** 10.1155/2017/1294913

**Published:** 2017-12-19

**Authors:** Jason K. Panchamia, David A. Olsen, Adam W. Amundson

**Affiliations:** Department of Anesthesiology and Perioperative Medicine, Mayo Clinic, Rochester, MN, USA

## Abstract

**Background:**

Ipsilateral phrenic nerve blockade is a common adverse event after an interscalene brachial plexus block, which can result in respiratory deterioration in patients with preexisting pulmonary conditions. Diaphragm-sparing nerve block techniques are continuing to evolve, with the intention of providing satisfactory postoperative analgesia while minimizing hemidiaphragmatic paralysis after shoulder surgery.

**Case Report:**

We report the successful application of a combined ultrasound-guided infraclavicular brachial plexus block and suprascapular nerve block in a patient with a complicated pulmonary history undergoing a total shoulder replacement.

**Conclusion:**

This case report briefly reviews the important innervations to the shoulder joint and examines the utility of the infraclavicular brachial plexus block for postoperative pain management.

## 1. Introduction

Total shoulder arthroplasty is a major surgical procedure, with the potential for severe postoperative pain, especially in the first 48 hours after surgery [1]. The interscalene brachial plexus block is considered the optimal regional anesthetic technique for postoperative analgesia in healthy patients after shoulder surgery. However, the major disadvantage of the interscalene block is the risk of ipsilateral phrenic nerve paralysis, with an incidence as high as 100% [2], depending on the volume, concentration, and location of local anesthetic administered. Consequently, hemidiaphragmatic paralysis depresses respiratory function, specifically decreasing forced vital capacity, forced expiratory volume in 1 second, and peak expiratory flow rates, which may be detrimental to patients with poor pulmonary reserve [3].

Given the increasing number of patients with preexisting pulmonary conditions undergoing shoulder surgery, current research is directed at diaphragm-sparing nerve block techniques. An example of these techniques is combining an infraclavicular brachial plexus block and a suprascapular nerve block [4, 5]. To date, there is limited literature regarding the analgesic efficacy of this combined technique. We report the successful application of a combined ultrasound-guided infraclavicular brachial plexus block and suprascapular nerve block in a patient with moderate-to-severe chronic obstructive lung disease undergoing total shoulder arthroplasty. The patient provided written consent to review and report this case.

## 2. Case Report

A 67-year-old man (American Society of Anesthesiologists physical status class IV; height, 170 cm; weight, 84.3 kg) was seen for a right reverse total shoulder arthroplasty. He had multiple comorbid conditions, including a history of a traumatic brain injury resulting in residual right-sided hemiparesis complicated by limb spasticity and neck contractures (making him wheelchair bound), poor functional status, and significant pulmonary disease. His pulmonary history included moderate-to- severe chronic obstructive pulmonary disease secondary to a 50-pack-year smoking history (Global Initiative for Obstructive Lung Disease Class C; forced vital capacity, 59%; forced expiratory volume in 1 second, 55%; diffusing capacity of the lungs for carbon monoxide, 53%), severe thoracic kyphosis resulting in restrictive lung disease, bronchiectasis complicated by impaired mucociliary clearance with mucus pooling notable on imaging studies, neuromuscular weakness in the setting of traumatic brain injury leading to poor respiratory effort, and obstructive sleep apnea necessitating continuous positive airway pressure therapy. The patient's baseline pain in his right shoulder was 9 out of 10 on a numeric pain rating scale (NRS), which he treated with scheduled acetaminophen and tramadol 75 mg per day.

Given the risk of hemidiaphragmatic paralysis after interscalene block, as well as the technical difficulty of performing nerve blocks above the clavicle because of the patient's neck contracture, the anesthesia team opted to perform a combined ultrasound-guided infraclavicular and suprascapular nerve block in the preoperative period. After appropriate monitoring and sedation, the ultrasound-guided infraclavicular block was performed via paracoracoid approach by visualizing the neurovascular bundle in a parasagittal plane just medial and inferior to the coracoid process. A 21-gauge, 100-mm insulated needle was advanced in-plane in a cephalad-to-caudad trajectory under direct visualization, with the needle tip positioned cephaloposteriorly to the axillary artery. A single injection of 15 mL of 0.5% bupivacaine with 1 : 200,000 epinephrine and 25 mcg of dexmedetomidine was administered, evaluating for a U-shaped spread, defined as local anesthetic distribution in a cephalad, posterior, and caudad position to the axillary artery, as described by Dingemans et al. [6].

An ultrasound-guided suprascapular nerve block, described by Harmon and Hearty [7], was performed by advancing the needle beneath the transverse scapular ligament into the suprascapular notch within the vicinity of the suprascapular nerve. A single injection of 10 mL of 0.5% bupivacaine with 1 : 200,000 epinephrine and 25 mcg of dexmedetomidine was administered.

The patient underwent the procedure supported, uneventfully, with general anesthesia and received a total of 100 mcg fentanyl, 10 mg ketamine, and 4 mg dexamethasone, all intravenously. During wound closure, the surgeon injected the incision site with 0.25% ropivacaine. The patient was successfully extubated and transported to the postanesthesia care unit, where he reported an NRS score of 0 and received no additional pain medication. He required minimal oxygen (2-L nasal cannula) and had an appropriate motor and sensory blockade in the expected infraclavicular distribution from the ipsilateral deltoid muscle to his fingers.

On the inpatient surgical unit, the postoperative pain regimen consisted of scheduled acetaminophen 1,000 mg every 6 hours and tramadol 25 mg every 6 hours as needed for breakthrough pain. In the first 20 hours postoperatively, the patient's NRS score remained 0, he did not receive any opioids, his pulmonary function was back to baseline with no additional oxygen requirement, and he continued to display motor blockade and sensory numbness of his right upper extremity, although he slowly regained motor function in his fingers. At 24 hours postoperatively, the patient's NRS score remained 0, he received 25 mg of oral tramadol for left arm spasticity pain, and his motor and sensory blockade resolved. At 27 hours postoperatively, the patient began to experience discomfort in his right shoulder, NRS score of 4, at which point he resumed his daily 75-mg tramadol pain regimen.

No adverse respiratory events or complications occurred throughout the patient's hospitalization. Although patients undergoing total shoulder arthroplasty at our institution are typically discharged on postoperative day 1, the patient was awaiting skilled nursing facility placement and was therefore discharged on postoperative day 2.

## 3. Discussion

The majority of the glenohumeral joint is innervated by the suprascapular nerve (C5-C6; originates from upper trunk of brachial plexus) and the axillary nerve (C5-C6; originates from posterior cord of brachial plexus). Furthermore, the shoulder joint and adjacent soft tissues receive minor contributions from the subscapular nerve (C5-C6; originates from posterior cord of brachial plexus), lateral pectoral nerve (C5-C6; originates from lateral cord of brachial plexus), and musculocutaneous nerve (C5–C7; originates from lateral cord of brachial plexus) [8]. The cutaneous innervation of the shoulder is supplied by the superficial cervical plexus (C1–C4).

An interscalene block performed at the level of the roots (C5–C7) or trunks (specifically upper trunk) of the brachial plexus, in combination with a superficial cervical plexus block, essentially allows for a complete analgesic technique to the shoulder joint. The phrenic nerve (C3–C5) and brachial plexus lie deep to the prevertebral fascia, thus local anesthetic administration after a brachial plexus block can result in medication “spilling” over onto the phrenic nerve. Kessler et al. [9] showed that the phrenic nerve and C5 nerve root are within 2 mm of each other at the level of the cricoid cartilage (also referred to C6 level). The distance between the phrenic nerve and brachial plexus increases approximately 3 mm for every centimeter caudal to the cricoid cartilage. Therefore, the risk of hemidiaphragmatic paralysis would be notably reduced, if not eliminated, if the local anesthetic injection were focused on the terminal nerves and associated articular branches of the brachial plexus, a distance considerably away from the phrenic nerve. Conversely, performing distal nerve blocks to minimize phrenic nerve blockade, such as the combined suprascapular and axillary nerve block approach, results in an incomplete analgesic technique. This would lead to suboptimal pain control due to the remaining unblocked minor neural contributors to the shoulder capsule (i.e., subscapular nerve, lateral pectoral nerve, musculocutaneous nerve, and superficial cervical plexus) [10]. Selective targeting of the posterior and lateral cords of the brachial plexus, in combination with suprascapular and superficial cervical plexus block, would provide improved postoperative analgesia by covering a greater part of the innervation to the shoulder joint. Tran et al. [4] stated that the combined infraclavicular plus suprascapular nerve block for shoulder surgery has been overlooked and forgotten. Our own literature search yielded only 1 case report from 2003 that described a combined infraclavicular plus suprascapular nerve block with nerve stimulator and high local anesthetic volumes to achieve surgical anesthesia in a patient with obstructive airway disease undergoing humeral head surgery [5].

Several considerations regarding the infraclavicular nerve block should be highlighted. First, there remains a potential concern for phrenic nerve paralysis after an infraclavicular nerve block. Petrar et al. [11] reported a 3% incidence of complete paralysis and a 13% incidence of complete or partial paralysis after a paracoracoid infraclavicular block entailing 30 mL of 0.5% ropivacaine injection for upper extremity surgery. This study used a large volume of local anesthetic to achieve surgical anesthesia before upper extremity surgery. It is important to distinguish local anesthetic volume and concentration necessary for surgical anesthesia versus postoperative analgesia. Classically, higher doses of local anesthetic (i.e., volume and concentration) are required to achieve surgical anesthesia, with consideration of providing complete anesthesia to the surgical site, along with enhanced sensory and motor block onset times. In comparison, peripheral nerve blocks intended solely for postoperative analgesia typically require lower local anesthetic doses. Furthermore, ultrasound guidance allows for a more targeted local anesthetic injection (i.e., to neural structures responsible for postsurgical pain) with a decrease in local anesthetic volume. In our case, the local anesthetic was injected in a cephaloposterior position to target mainly the posterior and lateral cords that provide innervation to the shoulder joint. In addition, we used a perineural dexmedetomidine/local anesthetic combination to potentially decrease the total dose of local anesthetic administered, while prolonging analgesia [12]. Further studies are necessary to elucidate the minimum effective volume to prevent hemidiaphragmatic paralysis while providing postoperative analgesia (as opposed to surgical anesthesia) for the infraclavicular block.

Second, the location of the infraclavicular brachial plexus block is important. The cords of the brachial plexus are labeled based on their position to the axillary artery. The infraclavicular block is commonly performed in the lateral infraclavicular fossa (also known as the* paracoracoid approach*). However, the cords in the lateral infraclavicular fossa can appear deep on ultrasonography, are difficult to visualize, and display variable anatomic positions around the axillary artery [13].

Fortunately, a single-injection technique of local anesthetic posterior to the axillary artery results in blockade of all 3 cords, even if the cords are not visualized [6, 13]. One of the challenges of a paracoracoid infraclavicular block is needle visualization, which becomes more difficult with increasing depth and extreme needle angulation. Abduction of the arm to 90° has been shown to decrease the distance from skin to the brachial plexus via ultrasound guidance [14]; however, performing abduction maneuvers would be extremely difficult in patients with severe shoulder and/or rotator cuff pathologic processes. Another approach to the infraclavicular block is the costoclavicular technique, which blocks the brachial plexus at the mid infraclavicular fossa, located under the midpoint of the clavicle. At this location, the cords of the brachial plexus are easier to visualize, the depth of the brachial plexus is superficial compared with the paracoracoid approach, and the 3 cords are reliably located lateral to the axillary artery in a compact space [15]. Given the satisfactory ultrasound imaging of the brachial plexus and the possible benefit of a more distal location from the phrenic nerve, we used the paracoracoid approach for this case. We cannot recommend one approach to the infraclavicular block over another, and further investigations are essential to determine the utility of either technique for postoperative pain control after shoulder surgery while minimizing phrenic nerve blockade.

In conclusion, knowledge of the anatomical innervation to the shoulder joint is critical in tailoring postoperative pain management and analgesic outcome expectations after shoulder surgery. The combined low-volume, ultrasound-guided, infraclavicular plus suprascapular nerve block effectively targets most of the neural innervations to the shoulder joint, thereby providing satisfactory postoperative analgesia, as demonstrated in this case report for a total shoulder arthroplasty. Future investigations should be directed at comparing the postoperative analgesic efficacy for total shoulder arthroplasty between ultrasound-guided interscalene blocks and the combination of infraclavicular plus suprascapular nerve blocks.

## Abbreviations

NRS: Numeric pain rating scale.

## Additional Points

*Implication Statement.* Phrenic nerve paralysis is a common adverse event after an interscalene brachial plexus block; it results in respiratory compromise, particularly in patients with pulmonary disease. Combined infraclavicular and suprascapular nerve blocks provide adequate analgesic coverage after shoulder replacement surgery while minimizing the risk of phrenic nerve blockade.
